# Dietary Inclusion Level Effects of Yoghurt Acid Whey Powder on Performance, Digestibility of Nutrients and Meat Quality of Broilers

**DOI:** 10.3390/ani13193096

**Published:** 2023-10-04

**Authors:** Vasileios V. Paraskeuas, Georgios Papadomichelakis, Ioannis P. Brouklogiannis, Evangelos C. Anagnostopoulos, Athanasios C. Pappas, Panagiotis Simitzis, Georgios Theodorou, Ioannis Politis, Konstantinos C. Mountzouris

**Affiliations:** 1 Laboratory of Nutritional Physiology and Feeding, Department of Animal Science, School of Animal Biosciences, Agricultural University of Athens, Iera Odos 75, 11855 Athens, Greece; v.paraskeuas@aua.gr (V.V.P.); gpapad@aua.gr (G.P.); gbrouk@outlook.com (I.P.B.); vagosanagn@gmail.com (E.C.A.); apappas@aua.gr (A.C.P.); 2 Laboratory of Animal Breeding and Husbandry, Department of Animal Science, Agricultural University of Athens, Iera Odos 75, 11855 Athens, Greece; pansimitzis@aua.gr (P.S.); gtheod@aua.gr (G.T.); i.politis@aua.gr (I.P.)

**Keywords:** acid whey, broilers, performance, meat quality, oxidative stability, digestibility

## Abstract

**Simple Summary:**

Greek strained yoghurt is a highly nutritious product with continuously growing production that leads to massive amounts of by-products such as acid whey. The large production of acid whey and the difficulties related to its processing make it a strong pollutant with costly environmental effects. Industry and academia are searching for sustainable applications to repurpose its use. Our research study examined the effects of yoghurt acid whey powder on broiler productivity and meat quality. Consequently, it did not adversely affect performance and enhanced meat quality by extending oxidative stability. In conclusion, yoghurt acid whey powder may be applied in broiler diets when added at 25 g/kg.

**Abstract:**

In recent years, the increasing demand for Greek strained yoghurt produced massive amounts of acid whey, which constitutes a major environmental pollutant. Whether yoghurt acid whey can be included in poultry diets is not known. The purpose of this study was to evaluate the effects of four dietary levels of yoghurt acid whey powder (YAWP) on the growth performance, nutrient digestibility, meat quality traits and oxidative stability. A total of 300 male 1-day-old Ross 308 broilers were assigned into four groups with five replicates of 15 broilers each: control-fed basal diet with no YAWP addition (WO) or basal diet supplemented with YAWP at 25 g/kg of diet (WA), 50 g/kg of diet (WB), or 100 g/kg of diet (WC). At the starter period, body weight and body weight gain were reduced after WB and WC treatments compared to the WO treatment. Breast meat oxidative stability was improved during refrigerated storage for 1 and 3 d in all YAWP treatments compared to control, while the WA treatment showed an improved oxidative stability after 6 and 9 d. The results suggest that YAWP inclusion at 25 g/kg of diet did not impair performance and extended the meat shelf life by reducing lipid oxidation rates.

## 1. Introduction

There are several types of whey such as acid whey and sweet whey, which except in the range of pH differ in proteins, lactose, lactic acid and Ca content [1]. Acid whey is a by-product derived from the manufacture of cream and cottage cheeses and from Greek strained yoghurt. The term acid whey comes from the low pH of whey, which ranges from 3.6 to 4.5 [2]. The Greek strained yoghurt is a highly nutritious product, with growing production reaching 195,510 tn in 2020 [3]. Yoghurt acid whey (YAW) is a dairy by-product that contains peptides and proteins, lactose, lactic acid, minerals such as calcium, potassium and phosphorus as well as vitamins [4]. However, the large amounts of YAW produced from the dairy industry in combination with its high biological oxygen demand (ranging from 52,400 to 62,400 mg/L) and organic matter make it a strong pollutant that must be either disposed of or repurposed to not cause costly effects on the surrounding ecosystems [3,5].

In recent years, dairy food industries have attempted to find sustainable applications to repurpose the use of large volumes of acid whey. These applications mainly occurred at their facilities to reduce transportation costs and range from isolating its valuable components to filtering it to reduce costs [3,6,7]. However, these endeavors of industry have not been successful due to compositional differences that affect the physicochemical characteristics of YAW’s separated compounds, and considering that the related science that supports these compositional effects on several YAW processing applications is not fully understood, the solutions of its disposal problem are still limited [5]. Moreover, the composition of YAW depends on the discrepancies of the environmental conditions during the process, which are reflected in the lactose concentration and the presence of minerals, organic acids, and pH [8]. Currently, if acid whey is not discarded, it is used as fertilizer on farmlands, as a source of energy in wastewater bioreactors, and in the production of valuable compounds via fermentation [3,9].

In the last few years, researchers have shown increasing interest in the investigation of the use of different whey types as poultry feed additives due to their potential health benefits that result from the fact that they contain valuable components [10]. Most studies have focused mainly on the use of sweet whey protein, which is a quickly absorbed protein source [11,12,13,14], and dried sweet whey powder [15,16,17,18,19,20] in poultry diets. Their dietary inclusion improved the broiler performance [11,13,14,15,16,18,19,20], digestibility of protein, fat and minerals [17,18], meat quality traits [13] and meat oxidative stability [12,13]. It must be noted that these effects depend on the composition of whey either in protein or dried powder applications as feed additives in broiler diets mainly due to the concentration differences in lactose and protein [18,20]. 

However, research on the effects of acid whey dietary addition in broiler diets is scarce. Only one study evaluated different dietary levels of low (40%) and high (78%) lactose acid whey powder on broiler performance, but the acid whey was derived from milk [21]. Finally, from a food perspective context, three papers have dealt with the determination of quality traits and oxidative stability of chicken meat after its marination in YAW [9,22,23]. Thus, the objective of the present study was to investigate for the first time the effects of four different dietary inclusion levels (0, 25, 50 and 100 g/kg of diet) of yoghurt acid whey powder (YAWP) on the broiler performance, digestibility of nutrients as well as meat quality characteristics and oxidative status.

## 2. Materials and Methods

### 2.1. Animal and Diets

A total of 300 male 1-day-old Ross 308 broilers were purchased from a commercial hatchery and vaccinated at hatch for Marek, Infectious Bronchitis and Newcastle Disease from a commercial hatchery. On arrival, broilers were randomly placed in 4 experimental treatments with 5 replicates per treatment consisting of 15 broilers each. All experimental treatments received a corn–soyabean meal basal diet as pellets, which was formulated according to Ross 308 nutrient requirements, and a 2-phase feeding plan was followed [1 to 10 days (starter period) and 11 to 35 d (grower-finisher period)]. The ingredients and the chemical composition of the experimental diets are presented in Table 1 and Table 2. The 4 experimental treatments were as follows: the first treatment (control) was fed with basal diet (WO) with no acid whey addition; the second treatment was fed basal diet containing yoghurt acid whey powder (YAWP) at 25g/kg of diet (WA), the third treatment was fed basal diet containing YAWP at 50g/kg of diet (WB) and the fourth treatment was fed basal diet containing YAWP at 100 g/kg of diet (WC). Yoghurt serum was derived mechanically after the fermentation of Greek-style strained yoghurt with Streptococcus thermophilus and Lactobacillus bulgaricus. The powder had moisture and ash concentrations of 4% and 11%, respectively. The YAWP contained 72% lactose, 8.5% galactose 6% lactic acid, and 5% protein as well as 24.7 g potassium, 18 g calcium, 14.4 g chloride, 6.6 g sodium, 6 g phosphorus, 1.7 g magnesium, 1.13 mg ferrum, and 0.48 mg copper per kg (Epirus Protein S.A., Ioannina, Greece).

Each treatment replicate was assigned to a clean floor pen (2 m^2^), and birds were raised on rice-hull litter. Birds had 24 h light during Day 1; then, they had 23 h light and 1 h dark until Day 7. From Day 8 to Day 10, the lighting program was set to 18 h light and 6 h dark. Throughout the experiment, feed and water were available ad libitum. Housing, management and care of the animals complied with the current European Union Directive on the protection of animals used for scientific purposes (EC 43/2007, EU 63/2010; Council of the European Union 2007, 2010). The experimental protocol was approved (protocol number: 51/21102021) by the Bioethics Committee of the Agricultural University of Athens (AUA), Greece.

### 2.2. Growth Performance Parameters

Broilers and feed remnants were weighed at d 10 and 35 on a pen basis. Growth performance parameters such as body weight gain (BWG), feed intake (FI), and feed conversion ratio (FCR) were determined for the starter (1–10 d) and intermediate period (11–35 d) as well as for the overall experimental period (1–35 d). The calculation of FCR was conducted according to the following equation: g FI/g BWG. Mortality was recorded daily.

### 2.3. Total Tract Apparent Digestibility of Nutrients 

The digestibility study was carried out after the growth performance study at 35 d using 5 birds per treatment transferred to cages with raised floors to enable excreta collection. Digestibility diets were the same as treatment diets for the grower-finisher period for WO, WA, WB and WC. The digestibility study had a 4-day pre-experimental adaptation period and a 3-day excreta collection period and was conducted according to Mountzouris et al. (2011) [24]. During the 3-day collection period, excreta from each cage were collected three times daily (i.e., with 8 h intervals) and stored in sealed bags at –20 °C. For total tract apparent digestibility, excreta collected per cage during the 3-day collection period were pooled and represented as one replicate (i.e., each treatment had five replicates). Feed and excreta samples were subsequently analyzed for dry matter, ash, ether extract and crude protein. Total tract apparent nutrient digestibility was measured by the following formula:

Total tract apparent digestibility of nutrients (%) = {[Ingested amount of nutrient (g) − Excreted amount of nutrient in excreta (g)]/Ingested amount of nutrient (g)} *×* 100.

### 2.4. Meat Quality Measurements

#### 2.4.1. pH24, Meat Color, Cooking Loss and Shear Force Value

Breast meat (right pectoralis major muscle) pH was determined 24 h after slaughter (pH24) by using a pH meter electrode (HI 99163 Meat pH Temperature Meter, Hanna instruments, Romania).

Meat color was determined on the surface of the broiler breast meat (right pectoralis major) after exposure for 30 min at room temperature (25 ± 2 °C) using a Miniscan XE (HunterLab, Reston, VA) chromameter set on the system color profile of lightness (L*), redness (a*) and yellowness (b*). The calibration of the instrument was conducted with a white and a black tile using illuminant D65 with 0° viewing.

Breast meat samples (right pectoralis major muscle) were dissected, weighed and placed in a thin-walled plastic bag and then were cooked in a water bath for 30 min at 80 °C. Afterwards, each sample was cooled under tap water and equilibrated at room temperature. Finally, the breast meat sample was weighed again for cooking loss (%) measurement. The determination of shear force value was conducted by cutting two 1.9 mm wide × 10 mm × 10 mm strips exactly from the center of breast meat samples and parallel to their muscle fibers. Then, samples were cut perpendicularly to the muscle fiber direction with a Zwick Testing Machine Model Z2.5/TN1S (Zwick GmbH and Co, Germany) which was equipped with a Warner–Bratzler shear [25]. The measurement unit of shear force values was N/cm^2^. All meat quality measurements were conducted at breast meat (right pectoralis major muscle) samples.

#### 2.4.2. Oxidative Stability

Oxidative stability determination was based on malondialdehyde (MDA) content. The MDA concentration was measured in the breast meat fillet sample (left pectoralis major muscle) from 10 chickens per treatment (i.e., 2 birds per replicate cage). Measurements were processed by using the selective third-order derivative spectrophotometric method after storage at 4 °C for 1, 3, 6 and 9 days and −20 °C for 30 and 60 days after slaughter in plastic sealed bags. In particular, 2 g of each meat sample (two samples per chicken) was homogenized in 8 mL aqueous trichloroacetic acid (TCA) (50 g/L) and 5 mL butylated hydroxytoluene in hexane (8 g/L), and then the solution was centrifuged for 5 min at 3000× *g*. Then, the top hexane layer was removed, and 1.5 mL of aqueous 2-thiobarbituric acid (8 g/L) was added to 2.5 mL from the bottom layer to be further incubated at 70° C for 30 min. This mixture was cooled under tap water and processed with third-order derivative (3D) spectrophotometry (Hitachi U3010 Spectrophotometer, Hitachi High-Technologies Corporation, Japan) in the range of 500–550 nm. The final concentration of MDA (ng/g wet tissue) was calculated as the height of the third-order derivative peak at 521.5 nm by referring to the standard calibration curve prepared using 1,1,3,3-tetraethoxypropane [26].

### 2.5. Statistical Analysis

The experimental data per treatment (WO, WA, WB and WC) were analyzed by using the general linear model (GLM)–ANOVA procedure of the SPSS for Windows statistical package program, version 27 (SPSS 17.0, Inc., Chicago, IL, USA). The statistically significant effects were further analyzed, and means were compared using Tukey’s honestly significant difference (HSD) multiple-comparison procedure. The linear and quadratic effects of dietary YAWP inclusion level were determined by using polynomial contrasts. Statistical significance was determined at *p* ≤ 0.05.

## 3. Results

### 3.1. Growth Performance Parameters

The results regarding the effect of YAWP dietary inclusion levels on broiler body weight (BW), body weight gain (BWG), feed intake (FI) and feed conversion ratio (FCR) are shown in Table 3. In the first 10 d, of the experiment, increasing the acid whey inclusion (YAWP) level reduced BW linearly (Plinear = 0.003), with treatments WB and WC producing lower (Panova = 0.011) BW compared with the control treatment WO. In addition, increasing the acid whey dietary level reduced the BWG in a linear (Plinear = 0.003) manner with treatment WB and WC showing lower (Panova = 0.013) BWG compared with treatment WO. Finally, no other significant differences were found regarding BW, BWG, FI and FCR during the starter (1–10 d), grower-finisher (11–35 d), and overall (1–35 d) period among the experimental treatments (Table 3).

### 3.2. Total Tract Apparent Digestibility of Nutrients

No significant effects of acid whey dietary supplementation were observed on total tract apparent digestibility (TTAD) of dry matter, ash, organic matter, ether extracts and crude protein, as shown in Table 4.

### 3.3. Meat Quality Measurements 

#### 3.3.1. pH24, Meat Color, Cooking Loss and Shear Force Value

Results for meat quality assessment are shown in (Table 5). The dietary inclusion of YAWP did not significantly affect (*p* > 0.05) pH24, lightness (L*), redness (a*), cooking loss or the shear force value of meat. On the other hand, the yellowness (b*) of meat was quadratically decreased (Pquadratic = 0.001) by YAWP dietary addition, with treatments WB and WC showing lower (Panova = 0.003) values for yellowness compared to that of the control treatment.

#### 3.3.2. Oxidative Stability

On day 1 of storage, the MDA values decreased in a linear (Plinear = 0.001) fashion, with treatments WA, WB and WC being lower (Panova = 0.002) than WO. Also, for 3 d storage, MDA displayed a linear (Plinear < 0.001) pattern of decrease with increasing YAWP level, with WA, WB and WC showing lower (Panova < 0.001) values than WO treatment. In addition, for 6 d storage, increasing the YAWP inclusion level resulted in a linear (Plinear < 0.001) pattern reduction with broilers treatment WA showing lower (Panova < 0.001) MDA values compared to WO. For 9 d storage, MDA values showed a linear (Plinear < 0.001) pattern of decrease increasing YAWP level, with treatment WA having lower (Panova = 0.005) values compared to WO treatment. Finally, MDA showed a linear (Plinear = 0.009) pattern of decrease with increasing YAWP inclusion level at 30 d of frozen meat storage (Table 6).

## 4. Discussion

The utilization of by-products as feed ingredients in poultry diets and their effects on broiler growth performance and health is a strategy that has been investigated in several studies [27,28,29]. Yoghurt acid whey (YAW) is a highly valuable dairy by-product with a composition that depends on the type of yoghurt from which it is derived, thermal processing of milk, storage conditions and more [3]. The applications of YAW in broiler diets are limited mainly due to its high (up to 70%) lactose content [21]. However, even though birds do not synthesize lactase to digest lactose, the latter can be fermented from bacteria in craw or their intestinal microbiota and as a result increase the production of volatile fatty acids in the gut and act as a natural prebiotic in poultry diets [20,30]. Moreover, the dietary inclusion of feed additives such as prebiotics has been reported to exert a modulating effect on lactic acid bacteria populations in the intestine of broiler chickens [31]. In addition, the protein, soluble vitamins and minerals contents make YAW a possible feed additive to promote broiler growth performance [21]. In general, the use of whey as a feed ingredient in poultry applications has been reported to induce contentious effects on broiler productivity [18,32]. These discrepancies are associated with YAW’s high compositional variability on lactose and protein [20,21]. 

In the present study, the dietary supplementation of yoghurt acid whey powder (YAWP) at 50 and 100 g/kg of diet reduced body weight (BW) and body weight gain (BWG) at the broiler starter period (1 to 10 d) without affecting feed intake (FI) and feed conversion ratio (FCR). These negative effects of YAWP addition on the broiler performance at 10 d of broiler age may be partially attributed to the higher inclusion levels of YAWP (5 and 10%) in broiler diets and consequently to the higher lactose concentration. It has been reported that the broiler lactose tolerance level is approximately 3.5%, and up to this point (in our case 3.6 and 7.2% lactose content at 50 and 100g YAWP/kg of diet, respectively), it may cause intestinal health and function issues with subsequent negative effects on broiler growth performance [32,33]. Moreover, these negative effects of YAWP addition on broiler performance when it was added at concentrations greater than 25 g/kg in broiler diets may be linked to the functional immaturity of the broiler digestive system and the intentional changes of intestinal microbiota populations at their early life stage [34].

At the intermediate (grower-finisher) (11 to 35 d) and overall (1–35 d) periods, growth performance was not affected from any of YAWP dietary inclusion levels. Although no differences were reported among dietary treatments for these periods regarding the overall BWG, FI values were higher and FCR values were lower than the respective performance objectives of the control Ross 308 male broilers in all cases. Specifically, for the 10 to 35 d period, BWG was higher by 11.5%, 9%, 10.3%, and 10.7%, while FI values were higher by 3.7%, 1.8%, 3.6%, and 2.8%, while FCR showed lower values by 6.8%, 6.8%, 6.1%, and 6.8% for the WO, WA, WB, and WC treatments, respectively, compared to the control Ross 308 performance objectives. As a result, overall (1–35 d), the performance parameters were also superior in the present experiment compared to the Ross 308 performance objectives, with BWG showing higher values by 11%, 8.6%, 9.2%, and 9.6%, FI showing higher values by 3.5%, 1.9%, 3.6%, and 2.4%, and FCR showing lower values by 6.4%, 5.7%, 5%, and 5.7% in the WO, WA, WB, and WC treatments, respectively. These growth performance responses were accompanied by low average experimental mortality rates at 0.44%, 0.92% and 1.33% for the starter, intermediate (grower-finisher) and overall period, respectively. The results of the present study suggest that YAWP dietary supplementation up to 10% did not impair the overall broiler growth performance and mortality. These findings could be linked with the properties of acid whey YAWP, which despite its low protein content contains lactose, lactic acid, a considerable amount of water-soluble vitamins, and a high abundance of minerals such as Ca [21].

To our knowledge, no studies exist concerning the effects of YAWP dietary addition in broiler diets on the total tract digestibility of nutrients. In addition, digestibility data for general dried whey powder application on ileal and total tract digestibility in broilers are scarce [18]. The current study has shown that the supplementation of YAWP at 2.5% (25 g/kg of feed), 5% (50 g/kg of feed) and 10% (100 g/kg of feed) did not affect the total tract apparent digestibility (TTAD) of dry matter, ash, organic matter, ether extracts and crude protein. In agreement with the aforementioned results, no significant effects regarding the protein and fat digestibility of dried whey powder application have been reported, but its dietary supplementation in broiler diets improved the digestibility of minerals such Ca and P [18]. Although the TTADs of Ca and P were not determined in this study, it is known that YAWP consists of considerable amounts of Ca and P, whose high digestibility could lead to higher mineral absorption and promote broiler growth [18,35]. This could be possibly related with the higher results for growth responses compared to the Ross 308 performance objectives in the present experiment. 

Meat quality traits and such as pH, color (lightness, redness, yellowness), cooking loss, and maximum shear force values are important aspects that directly affect the desire of consumers to purchase a meat product. In the present study, YAWP addition did not affect any of these measurements except breast meat yellowness, which was reduced when YAWP was supplemented at 50 and 100 g/kg of diet in broiler diets. In agreement with these results, the dietary addition of whey protein as a feed additive in broiler diets at 0, 0.15, 0.20, and 0.25% of diet improved all parameters related to meat color, and it did not significantly affect meat tenderness, water-holding capacity and cooking loss % [13]. The only studies from a food perspective context concerning the effects of YAW on meat quality traits are related to its usage in the marination of broiler chicken meat [9,22,23]. Using YAW in the marination of breast chicken meat has been reported to exert positive effects on juiciness [22], shear force values [9,22,23], redness (reduction) and yellowness (increase) [22]. An important factor for the explanation of these effects of YAW in broiler breast meat quality traits in vivo or after marination might be the large variability of its composition regarding lactose, lactic acid and calcium [5].

Our results revealed that the lipid oxidation of breast meat was delayed after YAWP supplementation at all dietary concentrations for 1 and 3 days, while the YAWP dietary inclusion at 25 g/kg retained higher oxidative stability after 6 and 9 days of refrigerated storage. It has been reported that the individual protein and peptides components of whey can exert antioxidant properties [1,36]. The antioxidant role of whey protein has been indicated in broilers fed 32 g/kg of diet for 42 days by reducing MDA in long-term breast meat refrigeration storage [12]. Moreover, increasing whey protein inclusion levels in broiler diets decreased MDA formation in the liver of 42-day-old broilers, confirming the antioxidant role of whey peptides and proteins [13]. However, no other literature studies were found testing the effects of YAWP inclusion levels on broiler breast meat oxidative stability.

## 5. Conclusions

Our study provides new data about the efficacy of YAWP dietary supplementation on broiler performance and meat quality traits and oxidative stability. It could be concluded that YAWP supplementation did not adversely affect the overall broiler performance and enhanced meat quality by extending its oxidative stability and hence refrigerated shelf life. The results of this work are inclusion level dependent, with the dietary addition of YAWP at 25 g/kg of broilers diet showing the best results. However, further research is needed in order to estimate the applicability of YAWP in broiler diets and better understand its effects on gut dynamics, gut integrity and health.

## Figures and Tables

**Table 1:** Composition of starter (1–10 d) basal diets.

Starter (0–10 d) Basal Diets	Treatments ^1^			
Ingredient %	WO	WA	WB	WC
Corn	49.9	47.65	44.56	38.37
Soybean meal	38.75	38.92	39.25	39.92
Soy oil	3.9	4.02	4.42	5.22
Corn gluten meal	2.5	2.5	2.5	2.5
Limestone	1.44	1.34	1.24	1.05
Mono-calcium phosphate	1.43	1.38	1.33	1.23
Salt	0.36	0.36	0.36	0.36
L-lysine HCL	0.34	0.34	0.34	0.34
DL-methionine	0.33	0.34	0.34	0.36
L-threonine	0.11	0.12	0.12	0.13
Vitamin premix	0.2	0.2	0.2	0.2
Mineral premix	0.2	0.2	0.2	0.2
Choline chloride 60%	0.09	0.09	0.09	0.09
Coccidiostat	0.05	0.05	0.05	0.05
Pelleting aid ^2^	0.4	0	0	0
Acid whey powder	0	2.5	5	10
Calculated composition				
AMEn (MJ/kg diet)	12.55	12.55	12.55	12.55
Dry matter %	88.26	88.35	88.54	88.91
Crude protein %	23.0	23.0	23.0	23.0
Ether extract %	6.17	6.22	6.53	7.16
Crude fiber %	3.46	3.43	3.38	3.29
Lysine %	1.44	1.44	1.44	1.44
Total sulfur amino acids (Met + Cys) %	1.08	1.08	1.08	1.08
Threonine %	0.97	0.97	0.97	0.97
Calcium %	0.96	0.96	0.96	0.96
Available phosphorus %	0.48	0.48	0.48	0.48
Sodium %	0.16	0.16	0.16	0.16

^1^ WO (basal diet no other additives), WA (basal diet containing 25 g YAWP/kg diet), WB (basal diet containing 50 g YAWP/kg diet) and WC (basal diet containing 100 g YAWP/kg diet). ^2^ Lignobond pellet binder (100% calcium lignosulphonate).

**Table 2:** Composition of intermediate (grower–finisher, 11–35 d) basal diets.

Intermediate (11–35 d) Basal Diets	Treatments ^1^			
Ingredient %	WO	WA	WB	WC
Corn	53.27	51.03	47.93	41.74
Soybean meal	36.63	36.8	37.14	37.8
Soy oil	5.89	6	6.41	7.21
Limestone	1.23	1.14	1.04	0.84
Mono-calcium phosphate	1.17	1.11	1.07	0.96
Salt	0.37	0.37	0.37	0.37
L-lysine HCL	0.16	0.16	0.16	0.16
DL-methionine	0.29	0.3	0.31	0.32
L-threonine	0.05	0.06	0.06	0.07
Vitamin premix	0.2	0.2	0.2	0.2
Mineral premix	0.2	0.2	0.2	0.2
Choline chloride 60%	0.08	0.08	0.08	0.08
Coccidiostat	0.05	0.05	0.05	0.05
Pelleting aid ^2^	0.4	0	0	0
Acid whey powder	0	2.5	5	10
Calculated composition				
AMEn (MJ/kg diet)	13.18	13.18	13.18	13.18
Dry matter %	88.13	88.23	88.42	88.79
Crude protein %	20.5	20.5	20.5	20.5
Ether extract %	7.96	8.01	8.33	8.95
Crude fiber %	3.38	3.35	3.3	3.21
Lysine %	1.23	1.23	1.23	1.23
Total sulfur amino acids (Met + Cys) %	0.95	0.95	0.95	0.95
Threonine %	0.83	0.83	0.83	0.83
Calcium %	0.83	0.83	0.83	0.83
Available phosphorus %	0.42	0.42	0.42	0.42
Sodium %	0.16	0.16	0.16	0.16

^1^ WO (basal diet no other additives), WA (basal diet containing 25g YAWP/kg diet), WB (basal diet containing 50 g YAWP/kg diet) and WC (basal diet containing 100 g YAWP/kg diet). ^2^ Lignobond pellet binder (100% calcium lignosulphonate).

**Table 3:** Effects of YAWP (yoghurt acid whey powder) dietary inclusion levels on broiler growth performance during the starter (1 to 10 d) and intermediate (11-35 d) growth periods and for the entire experiment (1-35 d).

Component ^1^	Treatments ^2^				Statistics ^4^			
	WO	WA	WB	WC	SEM ^3^	P_anova_	P_linear_	P_quadratic_
**BW**								
1 d	48.1	48.1	48.1	48.0	0.23	0.907	0.575	0.675
10 d	355.6 ^b^	351.6 ^ab^	337.5 ^a^	339.8 ^a^	5.54	0.011	0.003	0.427
35 d	2708.8	2651.5	2664.5	2675.1	28.75	0.264	0.347	0.115
**BWG**								
1–10 d	307.5 ^b^	303.5 ^ab^	289.3 ^a^	291.8 ^a^	5.63	0.013	0.003	0.427
10–35 d	2353.2	2299.9	2327.1	2335.3	28.85	0.348	0.776	0.151
1–35 d	2660.7	2603.3	2616.4	2627.1	28.72	0.263	0.349	0.113
**FI**								
1–10 d	300.2	301.8	291.2	290.0	7.73	0.337	0.113	0.809
10–35 d	3214.2	3155.2	3213.2	3186.6	44.73	0.525	0.863	0.614
1–35 d	3514.4	3456.9	3504.4	3476.7	47.34	0.616	0.666	0.662
**FCR**								
1–10 d	0.98	0.99	1.00	0.99	0.013	0.233	0.142	0.151
10–35 d	1.37	1.37	1.38	1.37	0.016	0.423	0.968	0.425
1–35 d	1.32	1.33	1.34	1.33	0.145	0.096	0.522	0.294

^1^ Data shown represent treatment means from n = 5 replicate floor pens for each treatment. ^2^ WO (basal diet no other additives), WA (basal diet containing 25 g YAWP/kg diet), WB (basal diet containing 50 g YAWP/kg diet) and WC (basal diet containing 100 g YAWP/kg diet). ^3^ Pooled standard error of means. ^4^ The statistical analysis tests the differences between treatments (ANOVA) and the linear and quadratic effect of whey inclusion levels (polynomial contrasts). Within the same row, means with different superscripts per treatment (a, b) differ significantly (*p* < 0.05).

**Table 4:** Effects of YAWP (yoghurt acid whey powder) dietary inclusion levels on total tract apparent digestibility coefficients of nutrients of 42 d old broilers.

Components ^1^	Treatments ^2^				Statistics ^4^			
	WO	WA	WB	WC	SEM ^3^	P_anova_	P_linear_	P_quadratic_
Dry Matter	75.3	76.5	77.6	76.6	2.11	0.755	0.478	0.456
Ash	44.4	47.7	51.2	49.5	5.21	0.608	0.270	0.505
Organic Matter	77.3	78.4	79.4	78.5	1.19	0.751	0.456	0.460
Ether Extracts	93.3	92.7	93.7	94.1	0.79	0.197	0.194	0.417
Crude Protein	69.1	68.6	71.0	66.3	3.35	0.589	0.587	0.387

^1^ Data represent treatment means from n = 5 replicate pens per treatment. ^2^ WO (basal diet no other additives), WA (basal diet containing 25 g YAWP/kg diet), WB (basal diet containing 50 g YAWP/kg diet) and WC (basal diet containing 100 g YAWP/kg diet). ^3^ Pooled standard error of means. ^4^ The statistical analysis tests the differences between treatments (ANOVA) and the linear and quadratic effect of whey inclusion levels (polynomial contrasts).

**Table 5:** Effects of YAWP (yoghurt acid whey powder) dietary inclusion levels on breast meat quality traits of 35 d old broilers.

Traits ^1^	Treatments ^2^				Statistics ^4^			
	WO	WA	WB	WC	SEM ^3^	P_anova_	P_linear_	P_quadratic_
pH	6.06	6.11	6.04	5.99	0.061	0.270	0.381	0.093
L*	58.07	55.98	56.23	55.49	1.355	0.263	0.206	0.231
a* ^5^	9.73	7.31	6.15	6.60	2.114	0.229	0.363	0.065
b*	20.26 ^b^	19.35 ^ab^	18.44 ^a^	17.88 ^a^	0.617	0.003	0.273	0.001
Cooking Loss (%)	11.02	12.40	11.29	11.57	1.045	0.587	0.252	0.708
Shear Force (N)	11.77	12.92	11.89	12.66	1.092	0.655	0.440	0.931

^1^ pH24 = pH 24 h after slaughter; L* = lightness; a* = redness; b* = yellowness. Cooking loss, % measurement. Shear force expressed on N/cm^2^. ^2^ WO (basal diet no other additives), WA (basal diet containing 25 g YAWP/kg diet), WB (basal diet containing 50 g YAWP/kg diet) and WC (basal diet containing 100 g YAWP/kg diet). Data represent treatment means from n = 10 broilers per treatment. ^3^ Pooled standard error of means. ^4^ The statistical analysis tests the differences between treatments (ANOVA) and the linear and quadratic effect of whey inclusion levels (polynomial contrasts). Within the same column, means with different superscripts per treatment (a, b) differ significantly (*p* < 0.05). ^5^ Data for a* = redness were transformed to ln.

**Table 6:** Effects of YAWP (yoghurt acid whey powder) dietary inclusion levels on breast meat oxidative stability of 35 d old broilers during storage (ng of malondialdehyde/g of meat).

Storage Time (d) ^1^	Treatments ^2^				Statistics ^4^			
	WO	WA	WB	WC	SEM ^3^	P_anova_	P_linear_	P_quadratic_
1	20.56 ^b^	12.38 ^a^	13.27 ^a^	14.25 ^a^	2.170	0.002	0.001	0.086
3	44.93 ^c^	24.33 ^a^	35.16 ^b^	34.27 ^b^	3.230	<0.001	<0.001	0.970
6 ^5^	58.48 ^b^	27.11 ^a^	38.15 ^ab^	48.72 ^b^	10.290	<0.001	<0.001	0.122
9	66.65 ^b^	45.86 ^a^	55.59 ^ab^	67.49 ^b^	6.417	0.005	<0.001	0.252
30	44.12	31.99	42.09	42.92	4.244	0.174	0.009	0.146
60	70.57	51.53	54.39	58.47	9.814	0.242	0.056	0.510

^1^ Measurements of malondialdehyde were implemented after storage at 4 °C for 1, 3, 6 and 9 days and −20 °C for 30 and 60 days after slaughter. ^2^ WO (basal diet no other additives), WA (basal diet containing 25 g YAWP/kg diet), WB (basal diet containing 50 g YAWP/kg diet) and WC (basal diet containing 100 g YAWP/kg diet). Data represent treatment means from n = 10 broilers per treatment. ^3^ Pooled standard error of means. ^4^ The statistical analysis tests the differences between treatments (ANOVA) and the linear and quadratic effect of whey inclusion levels (polynomial contrasts). Within the same column, means with different superscripts per treatment (a, b, c) differ significantly (*p* < 0.05). ^5^ Data for malondialdehyde after storage at 4 °C for 6 days were transformed to ln.

## Data Availability

The data analyzed in this study are available from the corresponding author on reasonable request.
